# Virome Variation during Sea Star Wasting Disease Progression in *Pisaster ochraceus* (Asteroidea, Echinodermata)

**DOI:** 10.3390/v12111332

**Published:** 2020-11-20

**Authors:** Ian Hewson, Citlalli A. Aquino, Christopher M. DeRito

**Affiliations:** 1 Department of Microbiology, College of Agriculture and Life Sciences, Cornell University, Ithaca, NY 14850, USA; cmd49@cornell.edu; 2 Department of Biology, Estuary and Ocean Science Center, San Francisco State University, Tiburon, CA 94920, USA; caquino6@mail.sfsu.edu

**Keywords:** densovirus, picornavirus, nodavirus, sea star wasting disease, asteroidea

## Abstract

Sea star wasting disease (SSWD) is a condition that has affected asteroids for over 120 years, yet mechanistic understanding of this wasting etiology remains elusive. We investigated temporal virome variation in two *Pisaster ochraceus* specimens that wasted in the absence of external stimuli and two specimens that did not experience SSWD for the duration of our study, and compared viromes of wasting lesion margin tissues to both artificial scar margins and grossly normal tissues over time. Global assembly of all SSWD-affected tissue libraries resulted in 24 viral genome fragments represented in >1 library. Genome fragments mostly matched densoviruses and picornaviruses with fewer matching nodaviruses, and a sobemovirus. Picornavirus-like and densovirus-like genome fragments were most similar to viral genomes recovered in metagenomic study of other marine invertebrates. Read recruitment revealed only two picornavirus-like genome fragments that recruited from only SSWD-affected specimens, but neither was unique to wasting lesions. Wasting lesion margin reads recruited to a greater number of viral genotypes (i.e., richness) than did either scar tissue and grossly normal tissue reads. Taken together, these data suggest that no single viral genome fragment was associated with SSWD. Rather, wasting lesion margins may generally support viral proliferation.

## 1. Introduction

Sea star wasting disease (SSWD) describes a condition that has been reported to affect Asteroidea since at least 1898 [1] and is associated with periodic mass mortality episodes, most recently during 2013–2014 [2]. The disease is pathognomic (i.e., has no distinguishing signs), where grossly abnormal specimens experience loss of turgor, abnormal limb twisting, epidermal lesions, body wall erosion, limb autotomy and in some cases death (reviewed in [3]). The cause of SSWD is currently unknown. Early work suggested association with a densovirus (the Asteroid ambidensovirus-1 [AaV-1]; also referred to as the “Sea Star associated Densovirus” [SSaDV]), and experiments which challenged healthy specimens with filtered tissue homogenates generated some SSWD signs [2,4]. However, subsequent work found that densoviruses, including AaV-1/SSaDV, occur in diverse asteroid taxa globally [5,6], and are highly prevalent within communities inhabiting the northeast Pacific [6] and northwest Atlantic [7] oceans. Recent investigations suggest that wasting response of asteroids to tissue homogenate challenge could generate via non-pathogenic means (i.e., through organic matter enrichment resulting in suboxic conditions through heterotrophic respiration) [8]. Other proposed mechanisms of wasting, including repeated [9] and monotonic [10] temperature excursions, high pCO_2_ conditions [11], and low precipitation [5] have all been hypothesized to influence SSWD. SSWD is not associated with any known eukaryotic microorganism nor bacterium [4,5,12,13,14]. Microbiome studies during wasting progression suggest a progressive enrichment of copiotrophic bacteria on surfaces and within tissues [8,12,14] but none appear distinct only to affected specimens.

RNA virome studies of SSWD to date have focused on snapshots of viral diversity comparing grossly normal to wasting affected specimens. For example, comparisons of RNA viral composition between disease states in *Pycnopodia helianthoides* found no RNA viral family consistently associated with SSWD-affected specimens that were absent from grossly normal individuals [2]. Similarly, RNA metavirome surveys of wasting *Pisaster ochraceus* discovered several candidate RNA viral genotypes [5]. However, subsequent qPCR studies and read recruitment of these genotypes failed to yield significant association with disease [5]. Hence, while RNA viruses occur in grossly normal echinoderm specimens and wasting-affected asteroid specimens, their potential roles in SSWD etiology are poorly resolved.

To further examine the role of viruses in SSWD, we observed time-course progression of wasting in *Pisaster ochraceus* during a concurrent study in which specimens wasted in the absence of external stimuli [8]. Histopathologic findings of SSWD indicate that affected body wall tissues experienced ulceration, cleft formation and coelomocyte aggregation, along with necrosis and body wall degradation [2,4,13,14]. We focused our study on body wall lesions, since these are the most conspicuous sign of SSWD, were the least subject to observer bias (c.f. deflation, limb curling), and lesions generally precede limb autotomy or body wall erosion. We hypothesized that viral etiologic agents would be would be present in wasting lesion margins, but absent in grossly normal tissues well away from lesions on the same individual, and absent in asteroid specimens that remained grossly normal during our study. Furthermore, we hypothesized that any SSWD-associated viral agent would be absent in physical scar margins in either SSWD-affected or grossly normal specimens, since these may be viruses that replicate during wound healing (c.f. cause pathogenesis). Our results demonstrate that there were no viral genome fragments which recruited reads only from wasting lesion margins in SSWD-affected asteroid specimens (i.e., all wasting lesion genome fragments also recruited reads from scar tissue or control tissues). Rather, we found a progressive enrichment of viral genotypes that recruited sequence reads in lesion margins over time in comparison to artificial scar and grossly normal tissues, suggesting their prominence in prior metaviromic surveys may have been independent of potential pathology.

## 2. Materials and Methods

### 2.1. Survey Design

**Figure 1:**
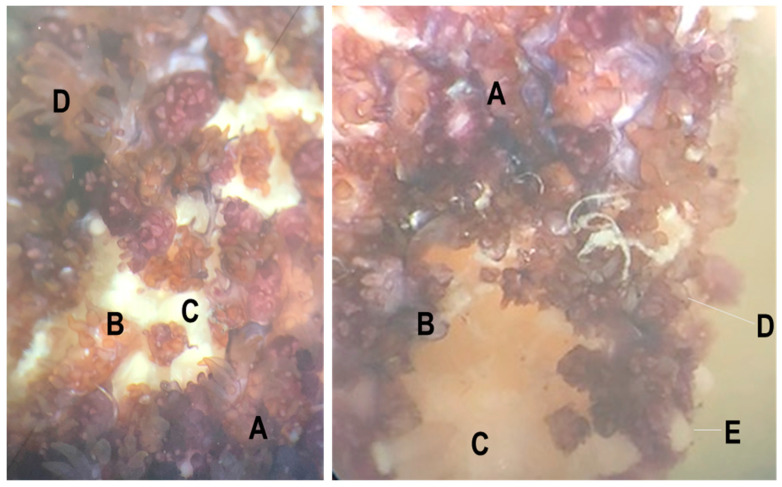
Gross examination of SSWD lesion on a *Pisaster ochraceus* specimen retrieved from Davenport, CA at the time of sampling for this survey. A = grossly normal tissue; B = lesion margin; C = lesion (underlying body wall tissues); D = papula and pedicellaria; E = paxilla (spine).

**Figure 2:**
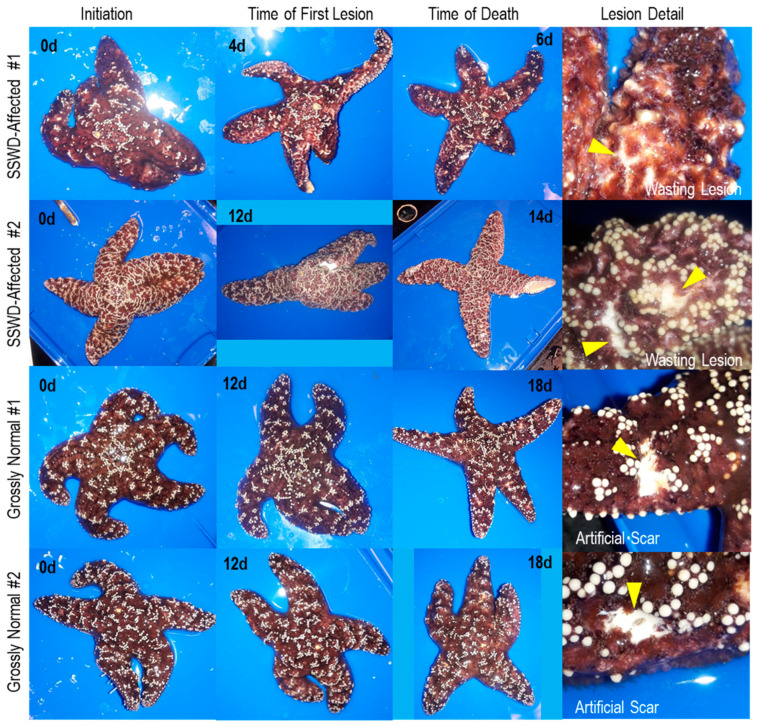
Gross changes in *Pisaster ochraceus* observed in specimens used in viral metagenome analyses over time, and detail (indicated by arrows) of wasting lesion and artificial scars sampled. The elapsed time is indicated on each panel.

Longitudinal survey of sea star wasting microbial ecology was performed in July–August 2018 and reported in separate studies focusing on associated bacteria [8]. We collected six *Pisaster ochraceus* specimens (mean mass 290 ± 54 g and ray length 11.2 ± 0.9 cm) from the intertidal zone at Davenport, CA, USA (37°1′19″ N, 122°12′56″ W) on 19 July 2018, which were transported in insulated coolers to the Long Marine Laboratory at UC Santa Cruz and housed in flow-through aquaria indoors in individual containers. After 48 h acclimation, a small scar (~5 mm long) was made on a single ray of each specimen using a sterile 4 mm biopsy punch. Individual asteroids were monitored daily for the presence of lesions (which were defined as non-focal loss of epidermal tissues revealing the underlying body wall; see Figure 1). After 72 h, artificial scar margin tissues (~3 mm × 2 mm) were collected using sterile 4 mm biopsy punches. New artificial scars were made on adjacent rays each day and sampled after each 24 h period. When wasting lesions were observed (the first wasting lesion was observed at 96 h), their margins were sampled following the same approach, and grossly normal tissues well away (>1 cm) from the lesion collected at the same time for comparison. A photographic summary of natural and wasting lesions is provided in Figure 2 and sampling schema in Figure S1. All tissue samples were placed into sterile 1.2 mL cryovials and immediately frozen in liquid N_2_ or in a −80 °C freezer. 

### 2.2. Viral Metagenome Preparation

We focused viral metagenomic analyses around four specimens - two that developed wasting lesions and died during the experiment (hereafter referred to as “SSWD-affected”), and two that remained grossly normal during the experiment (Table 1). Initial samples (taken at 0 h) were prepared for viral metagenomics from all four specimens. Viral metagenomes from SSWD-affected specimens were prepared from wasting lesion margin, artificial scar margin, and control tissue samples away from artificial scars and wasting lesions at the time of lesion genesis (96 and 288 h for wasting-affected specimens #1 and #2, respectively). In addition, viral metagenomes were prepared from wasting lesion margin samples taken from the SSWD-affected specimens at the time of death. Viral metagenomes were prepared from the two grossly-normal specimens at 0 h, and at 432 h from artificial scar margin tissues and tissue samples collected away from artificial scars.

Tissue samples were prepared for viral metagenomics targeting RNA viruses as described previously [15,16,17]. In addition to RNA viruses, we also examined densoviruses (i.e., ssDNA) viruses since they are also captured in libraries prepared using this approach [5]. Briefly, tissue samples were homogenized in 2 mL of 0.02 µm-filtered PBS by bead-beating (Zymo Bead Beaters; Zymo Research Irvine, CA, USA), briefly centrifuged at 3000× *g* for 30 s to remove large particulate matter, and then filtered through 0.2 µm filters (Durapore; MilliporeSigma, Burlington, MA, USA) to remove cell debris. The resulting concentrate was treated with DNAse I (5 U; Thermo Fisher Scientific, Waltham, MA, USA), RNAse One (50 U; Promega, Madison, WI, USA) and Benzonase (250 U; Sigma-Aldrich, St. Louis, MO, USA) for 3 h at 37 °C to remove co-extracted free nucleic acids, before arresting enzyme activity with 50 µM virus-free EDTA. Viral nucleic acids were extracted using the Zymo Viral RNA kit, before amplification using the TransPlex Whole Transcriptome Amplification kit (Sigma Aldrich). Resulting products were electrophoresed and quantified by Pico Green fluorescence. Samples were then submitted to Biotechnology Resource Center at Cornell University, where libraries were sequenced on two lanes of Illumina MiSeq (2 × 250 bp paired-end; San Diego, CA, USA) after TruSeq PCR-free library preparation. Sequence libraries are available at NCBI under BioProject PRJNA637333 and SRA accessions SRR11931172- SRR11931187. 

### 2.3. Bioinformatic Processing

Sequence libraries were initially trimmed for adapters and quality (*N* < 0.5) using the CLC Genomics Workbench 4.0 (CLC Bio, Aarhus, Denmark). We used an assembly-read mapping approach to examine the presence and absence of viral genome fragments between libraries. First, a global assembly of all 10 samples from SSWD-affected specimens (Table 1) was performed using the CLC Genomics Workbench 4.0 (Qiagen) using a minimum overlap of 0.5 and similarity of 0.8. The resulting contig spectra was aligned against several boutique databases of RNA viruses, encompassing genomes and proteins of invertebrate viral groups, by BLAST [18,19] as described elsewhere [17]. These boutique databases comprised viral genomes and proteins assembled from NCBI using the following word searches (accessed October 2018): all RNA viral library using tBLASTx [18] (search term “RNA Virus”); Mononegavirus proteins (search term “mononegaviruses”) by BLASTx; Picornavirus RNA-dependent RNA polymerase (RdRp) proteins (search term “RdRp AND picornavirus”) by BLASTx; invertebrate RNA viral proteins (search term “invertebrate AND RNA viruses”) by BLASTx; Flavivirus proteins (search term “flavivirus”) by BLASTx; Coronavirus proteins (search term “coronavirus”) by BLASTx; And nodavirus proteins (search term “nodavirus”). Since ssDNA viruses are also retrieved in RNA viromes [5], and since densoviruses had previously been associated with asteroids elsewhere [2], we also aligned contig spectra against densoviral genomes and proteins (search term “desovirus”) by tBLASTx and BLASTx respectively. Sequence matches against any of these databases at an *E*-value < 10^−20^ were further aligned against the non-redundant (nr) library at NCBI by BLASTx, and contigs discarded if they matched known bacterial or eukaryote proteins at a higher percentage and lower *E*-value than viruses. Contigs which had <5× coverage were discarded from analysis, and contigs were inspected for potential chimeras by coverage long contig lengths (see Figure S2 for coverage plots). 

The resulting contig spectra (here termed “viral genome fragments”) were then subject to read recruitment independently across the 10 SSWD-affected specimen libraries and the additional 6 libraries from grossly normal specimens, where we considered a successful detection as recruiting ≥2 reads. Read recruitment was performed using the native algorithm in the CLC Genomics Workbench 4.0, using parameters of minimum overlap 0.2 and minimum similarity of 0.95. Because we did not standardize template nucleic acid quantities nor total viral abundance in amplification reactions, we were unable to gain quantitative insight into representation during wasting progression [20]. Hence, our work focuses only on the presence of viral genome fragments and their presence by read recruitment between viromes. Viral genome fragment sequences are available at NCBI under accessions MW073776-MW073820.

### 2.4. Phylogenetic Analyses

The phylogenetic placement of viral contigs was investigated by performing alignments of viral open reading frame (ORF) translated amino acids against close relatives retrieved at NCBI using the progressive alignment algorithm of the CLC Genomics Workbench 4.0 (Qiagen, Hilden, Germany) and using a gap open cost of 10 and gap extension cost of 1. The resulting gapped alignment was trimmed to an overlapping region. Two methods were used to perform phylogenetic reconstructions based on these alignments. First, trees were constructed using Jukes-Cantor distance and by Neighbor-Joining in the CLC Sequence Viewer 8.0. Second, trees were constructed using Maximum Likelihood, using the Poisson model of substitution, uniform rates among sites, and using all gaps/missing data, and Nearest-Neighbor-Interachange (NNI) heuristic model using MEGAX (version 10.1.8; [21]). For both analyses, trees were bootstrapped with 1000 replications and presented as unrooted dendrograms.

### 2.5. Statistical Analyses

We analyzed the presence/absence of viral genome fragment recruits between: grossly-normal and SSWD-affected specimens; between initial, wasting lesion, artificial scar, and control tissues away from lesions/scars; and between timepoints on the same individual at the time of lesion genesis and death by performing Fisher’s Exact test to address the hypothesis that the recruitment to viral genome fragments in each specimen condition, tissue types, and sample time were independent. The variation in read recruitment richness between tissue types (initial, grossly normal at time of lesion genesis + conclusion of study, artificial scar at time of lesion genesis + conclusion of study, and wasting lesion at time of genesis + death) was examined by performing pairwise Student’s *t*-tests with Bonferroni correction to account for Type II error in multiple comparisons. All analyses were performed in XLStat (AddinSoft GmBH, Paris, France). 

## 3. Results and Discussion

Our temporal survey of virome composition during SSWD progression further advances understanding that echinoderms, including asymptomatic individuals, harbor rich viral assemblages but that there is little association between specific viral genotypes and wasting signs [5,6]. Specimens that wasted in our survey experienced loss of turgor, followed by the appearance of epidermal lesions. Microscopic investigation (Figure 1) showed that these were due to loss of epidermal tissues exposing the underlying body wall. Grossly, lesion margins were unremarkable and were not melanized. Between the time of first lesion appearance and animal death, SSWD-affected specimen #2 autotomized a ray, while SSWD-affected specimen #1 experienced body wall erosions that allowed internal organs (presumably pyloric caeca) to protrude. Wasting lesions were not grossly distinct to artificial scars by visual inspection (Figure 2).

### 3.1. Description of Viruses Recovered in Viral Metagenomes 

Viral metagenomes prepared from 16 samples (Table 1) generated a total of 48,316,171 reads. Assembly of the 10 SSWD-affected specimen libraries (35,436,533 reads) resulted in 96,778 contigs (total reads assembled were 21,231,303 [60% of total reads]; average contig length was 922 nt (N50 was 910); coverage 49.0 for entire assembly and 23.5 for viral contigs). Of these, only 24 matched RNA or densoviral viral proteins or genomes by BLAST alignment (i.e., viral genome fragments) at ≥5× coverage. The remaining sequence space likely constitutes host or cellular microbiome material or bacteriophage which normally comprise a large proportion of host-associated metaviromes [17]. It is also possible that the remaining sequence space represents viral genomes which are not homologous with those represented at NCBI. Next, we recruited these against each of the 10 symptomatic libraries separately, as well as to 6 grossly normal specimen libraries. All but one viral contig recruited against >1 specimen in any tissue type, suggesting that most viruses inhabiting asteroid tissues are cosmopolitan between individuals within the *Pisaster ochraceus* population at the time of sampling. Viral contigs meeting these criteria were mostly similar to *Picornavirales* (*n* = 12 contigs), with fewer matches to *Piccovirales* (*Parvoviridae*; *Densovirinae*; *n* = 9 contigs), *Nodamuvirales* (*Nodaviridae; n* = 2 contigs), and *Sobelivirales* (*Solemoviridae; n* = 1 contig). 

**Figure 3:**
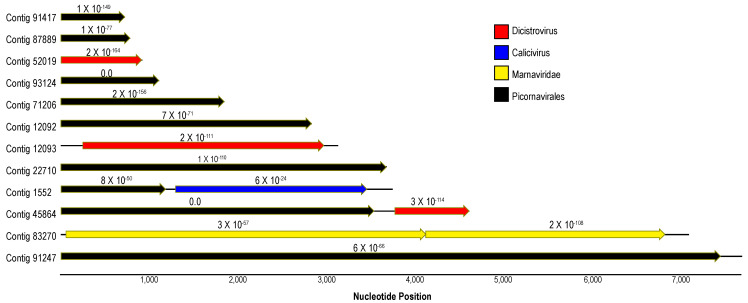
Maps of contiguous sequences matching *Picornavirales* recovered in this survey of *Pisaster ochraceus*. Contigs were annotated based on BLASTx (*e*-value < 1 × 10^−20^) against the non-redundant database at NCBI. The color of arrows (open reading frames) indicates the taxonomic identity of their best matches. Numbers above the ORFs indicate the *e*-value of BLAST results. The total contig lengths are indicated by solid lines running through and between ORFs.

**Figure 4:**
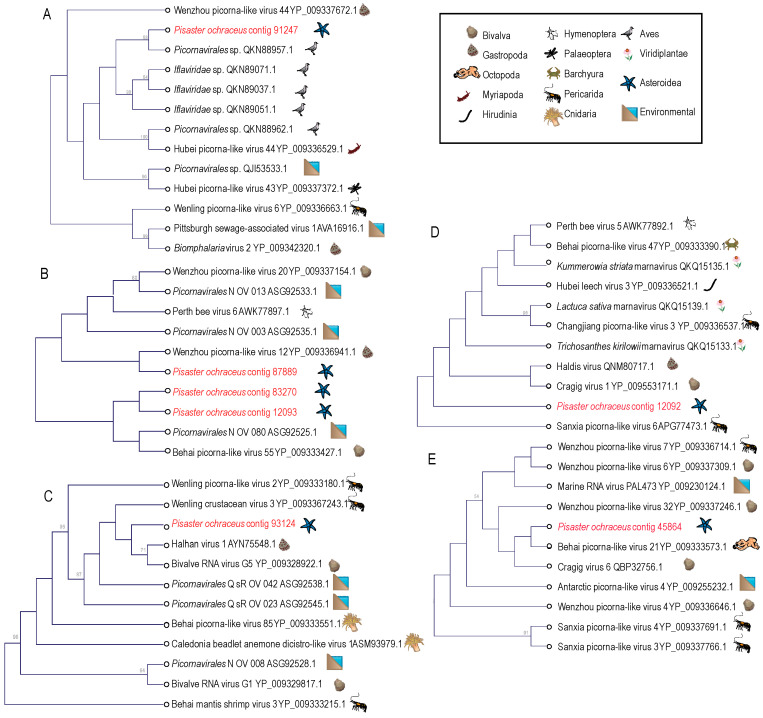
Phylogenetic representations of *Picornavirales*-like genome fragments recovered from *Pisaster ochraceus*. The cladograms were constructed based on a 98 amino acid alignment of the RNA dependent RNA polymerase gene (**A**), a 187 amino acid alignment of the rhv-like capsid domain (**B**), a 160 amino acid alignment of the RNA helicase domain (**C**) and 193 amino acid of the RNA dependent RNA polymerase gene (**D,E**) and performed separately for overlapping regions including best matches at NCBI. Trees were constructed by Neighbor Joining and based on Jukes-Cantor distance in the CLC Genomics Workbench 4.0 (Qiagen). Bootstrap values >50% (based on 1000 iterations) are indicated above nodes. The host identity is indicated by symbols next to branch labels. An additional phylogenetic representation of each tree based on maximum likelihood is presented in Figure S3.

Picornaviruses (+ssRNA) feature prominently in most host-associated virome surveys [22,23,24,25,26,27,28,29,30,31,32,33] and are ubiquitous in marine plankton as free particles [34,35,36,37,38,39,40]. Of the 12 picornavirus-like genome fragments recovered in this survey, three matched most closely to dicistroviruses (*Dicistroviridae*), one to bacillarnaviruses (*Marnaviridae*), and one to caliciviruses (*Caliciviridae*) by BLASTx (Figure 3). Furthermore, contigs clustered with iflaviruses (*Iflaviridae*; contig 91247) and marnaviruses (*Marnaviridae*; contig 12092) by phylogenetic analyses (Figure 4). Picornavirus-like genome fragments mostly matched viral genomes recovered from transcriptomic surveys of marine invertebrates (mollusks and crustacea) [22,41] and picoeukaryotes [42]. Picornaviruses have been previously recovered from asteroids [2,5] and holothurians [17]. Since several genome fragments recovered in this survey matched most closely picornaviruses recovered from protists [42], it is possible that they were associated with protozoa associated with wasting tissues. Previous work has revealed the presence of presumably fungal viruses and a wide richness of protistan rRNAs in metagenomes prepared from material purified from <0.2 µm filtered tissue homogenates [17]. Picornaviruses have been recovered in stressed marine metazoa [43,44], cause mortality in protists (e.g., diatom viruses [45,46]) and disease in marine arthropods (e.g., Taura syndrome virus [47]). However, the wide diversity of picornaviruses recovered from grossly normal specimens in field surveys [22] suggests that their role in disease, especially in mass mortality settings, is unclear for most hosts.

**Figure 5:**
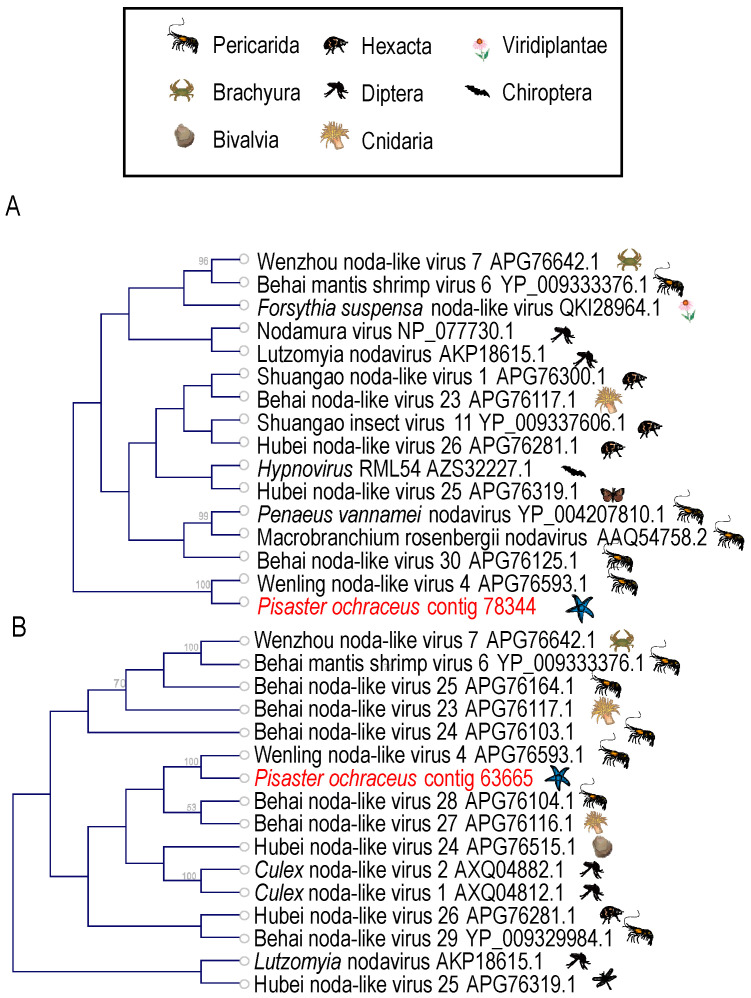
Phylogenetic representations of *Nodamuvirales*-like genome fragments recovered from *Pisaster ochraceus*. The trees were constructed based on a 101 amino acid (**A**) and 559 amino acid (**B**) alignments of the RNA dependent RNA polymerase gene of the nodavirus RNA1 genome fragment including best matches at NCBI. Trees were constructed by Neighbor Joining and based on Jukes-Cantor distance using CLC Genomics Workbench 4.0 (Qiagen). Bootstrap values >50% (based on 1000 iterations) are indicated above nodes. The host identity is indicated by symbols next to branch labels. An additional phylogenetic representation of each tree based on maximum likelihood is presented in Figure S5.

Nodaviruses (+ssRNA) represent significant pathogens of marine vertebrates (reviewed in [48,49]) and invertebrates [50] and are frequently recovered in grossly normal marine invertebrates [51,52,53,54]. Two viral contigs matching most closely nodaviruses by BLASTx (Figure S4) were recovered in our survey. Phylogenetic analyses placed these most similar to nodaviruses recovered from cnidarians, nematodes and arthropods as part of transcriptomic viral discovery efforts [22] (Figure 5). Nodavirus-like genome fragments were identified in the asteroid *Pycnopodia helianthoides* [2] and were present in wasting-affected *Pisaster ochraceus* libraries prepared from the Olympic National Park in 2013 (NCBI BioProject SAMN15704856; tBLASTx *e* < 1 × 10^−20^) [5], however were absent in a RNA viral metagenomic survey of holothurians [17]. Our observation of nodavirus genome fragments in asteroids extend the known host range of the *Nodamuvirales*.

**Figure 6:**
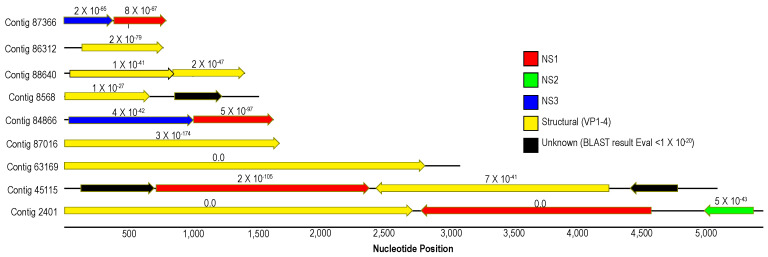
Map of densovirus-like genome fragments recovered from *Pisaster ochraceus* from Davenport, CA in July 2018. The length of contig is given by the solid black line running through open reading frames (ORFs; indicated by arrows). The color of arrow indicates the top BLASTx match to the non-redundant database at NCBI, and *e*-value of the match given above each ORF.

**Figure 7:**
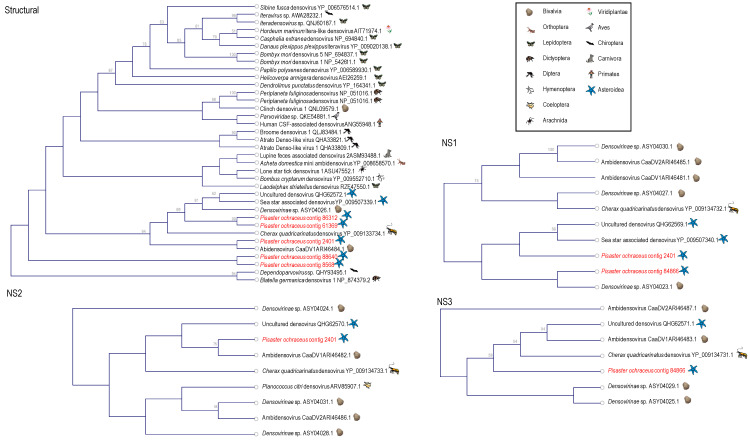
Phylogenetic representations of *Densovirinae*-like genome fragments recovered from *Pisaster ochraceus*. The cladorgrams were constructed based on: a 103 amino acid alignment of the structural (coat protein) gene; a 83 amino acid of the non-structural 1 (NS1) gene; a 112 amino acid of the NS2 gene; and a 111 amino acid of the NS3 gene. Phylogenetic representations include best matches by BLASTx against the non-redundant database at NCBI. Trees were constructed by Neighbor Joining and based on Jukes-Cantor distance using the CLC Genomics Workbench 4.0 (Qiagen). Bootstrap values >50% (based on 1000 iterations) are indicated above nodes. The host identity is indicated by symbols next to branch labels. An additional phylogenetic representation of each tree based on maximum likelihood is presented in Figure S6.

Densoviruses (*Piccovirales*; *Parvoviridae*, ssDNA) have been previously recovered from arthropods (reviewed in [55,56]), mollusks [57,58], a tunicate [59] and echinoderms [2,5,6,7]. Densoviruses are associated with mussel [58] and asteroid ([2] but see [6]) mass mortality. Contigs matching densoviral proteins and genomes recovered in this survey bore primarily structural ORFs and ORFs bearing the non-structural (NS; i.e., replication-associated protein) 1 regions, with fewer NS2 and NS3 regions (Figure 6). Genome fragments bearing both structural and non-structural regions (*n* = 3) bore ambisense genome architecture, suggesting these belonged to the *Ambidensovirus* genus to which almost all known marine invertebrate densoviruses belong [2,6,7,57,60]. Alignment of structural (coat protein) placed detected densovirus genome fragments within the ambidensovirus genus (Figure 7). Importantly, we did not recover any genome fragment bearing >85% nucleotide identity to Asteroid ambidensovirus 1 (i.e., SSaDV).

We also recovered a genome fragment matching a sobemovirus (*Solemoviridae*; Rice Yellow Mottle Virus NCBI CAE81305.1; BLASTx against nr database *e*-value = 1 × 10^−35^) [61]. Sobemoviruses cause a wide range of plant diseases, including mottles and mosaics (reviewed in [62]). This virus is unlikely to infect echinoderm cells and instead likely infected a microbiome constituent.

### 3.2. Analysis of Virome Association with Sea Star Wasting Disease

**Figure 8:**
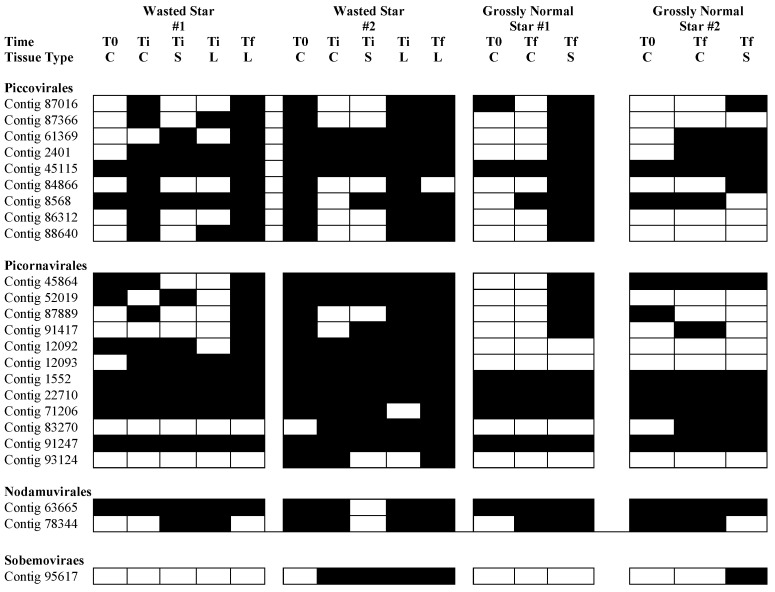
Heat map of viral contig read recruitment across all libraries in non-wasted and wasted asteroids. Dark cells = viral contig recruited reads from library, white cells = viral contig did not recruit reads from library. T0 = initial sample, Ti = time of lesion formation, Tf = experiment terminaton. C = grossly normal tissue, S = artificial scar tissue, L = wasting lesion margin. Phylogeny determined by family-level assignment based on nearest relative match (BLASTx) against non-redundant (nt) database at NCBI.

Of 24 viral genome fragments recovered in this survey, all recruited reads from SSWD-affected asteroids, but only 21 genome fragments recruited reads from grossly normal specimens (Figure 8). All genome fragments recruited reads from all tissue types. Only two genome fragments (contigs 12092 and 12093), both *Picornavirales* (putatively assigned to *Dicistroviridae*) recruited reads only from SSWD-affected specimen libraries (Table 2). However, both genome fragments did not recruit uniquely to wasting lesion margin libraries and also recruited reads from artificial scar margin and control tissue libraries. Recruitment to contigs 2401 (*Densovirinae*) and 12093 (*Picornavirales*) were significantly associated with time of sampling (i.e., mostly present in later samples), but also recruited reads from both grossly normal specimens and SSWD-affected specimens. Hence, our data do not support association between any viral genome fragment recovered in this survey and SSWD since none was unique to either wasting lesion margins or to SSWD-affected specimens. We cannot discount the possibility that viruses that fell below our threshold for detection (<5× coverage) or distant viruses not retrieved in our approach may occur in lesion margins. Our expectation was that any virus associated with wasting would be observed exclusively in lesion margins and likely at high viral load (note we cannot quantitatively compare libraries, but our expectation is that abundant viruses would comprise a much larger proportion of virome sequence space than less abundant viruses). Hence, possible viruses that were not observed via our approach are unlikely to be intimately associated with asteroid wasting.

The role of viruses in asteroid wasting etiology has been controversial. Early association between the Sea Star associated Densovirus (Asteroid ambidensovirus 1; AaV-1 [2]) was not supported by subsequent work [5]. The discrepancy between studies was attributed to inaccurate primer design on investigation outset (providing false positives as a result of a background of ambient densovirus strains), the low numbers of individuals sampled, and presence of AaV-1 in asymptomatic individuals. Further investigation of densoviruses in northwest Atlantic Ocean asteroids revealed the presence of persistent infection by a related strain of AaV-1 (Asteroid ambidensovirus 2) [7], and densoviruses have been recovered from asteroid tissue metagenome surveys elsewhere [5], suggesting that densoviruses may be common constituents of the asteroid microbiome. Of the 9 densovirus genome fragments recovered in the survey presented in this work, all recruited reads from >1 specimen, and six were detected in all four specimens. These results illustrate that densoviruses may be cosmopolitan within *Pisaster ochraceus* populations.

**Figure 9:**
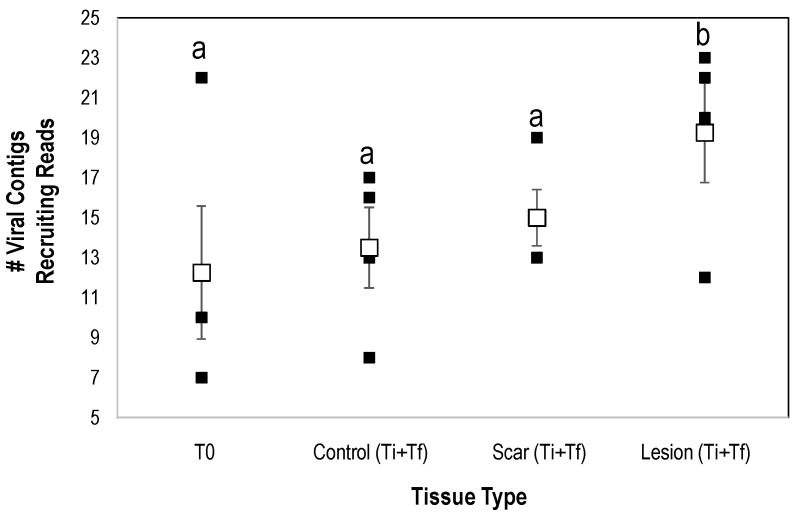
Richness of viral genome fragments recruiting reads from SSWD-affected and grossly normal tissues in viral metagenomes prepared from *P. ochraceus* during temporal study of wasting. Significance (a,b) determined by Student’s *t*-test (*p* < 0.008, df = 4 with Bonferroni correction for 6 tests). T0 = initial sample, Ti = time of first lesion appearance, Tf = experiment termination. Open squares represent mean (error bars = SE).

Interestingly, the number of viral genome fragments recruiting from natural lesion libraries was significantly greater (*p* < 0.008, Student’s *t*-test, df = 4) than both artificial lesion and control (i.e., away from lesions on SSWD-affected specimens) tissues (Figure 9). Previous work has noted taxonomic variation in host-associated viral communities in response to stress. For example, Laffy et al. [63] examined the impacts of thermal stress on the sponge *Rhopaloiedes odorabile* and observed the proliferation of Calimoviruses and Retroviruses relative to controls. Additionally, Grasis et al. [64] noted that the greatest viral diversity was observed in heat-stressed *Hydra* spp. when compared to controls. Vega Thurber et al. [65] noted an increase in herpesvirus-like sequences in stressed corals. While it is tempting to ascribe this result to enhanced susceptibility to opportunistic pathogens in compromised cells, it is more likely that our observation relates to factors affecting viral replication of normally asymptomatic viral infections. Viral replication in affected tissues is a complex interaction with intracellular properties and environmental cues. All viral groups detected in this study, including the *Densovirinae*, replicate in actively dividing host cells. Hence, the increase in viral richness in affected tissues, which were not grossly hyperplastic (i.e., did not display gross signs of rapid cell division), is surprising. Wound repair would presumably be associated with an increase in gene transcription in affected tissues, which in turn cause rapid replication of viruses in infected cells. Recently we observed that SSWD is associated with a proliferation of copiotrophic bacteria near and on their respiratory surfaces, concomitant with and followed by the presence of facultative and strictly anaerobic bacterial taxa, which may indicate suboxic conditions at the animal-water interface and within tissues [8]. O_2_ tension in cells triggers production of many viruses [66]. For example, flaviviruses and nucleocytoplasmic large DNA viruses use hypoxia inducible factors (HIF; genetic switches and genes that activate under hypoxic conditions), to stimulate production [67,68,69,70,71,72]. Hence, it is possible that suboxic stress may influence the proliferation of viruses in SSWD-affected asteroids.

The greater richness of viral genome fragments that recruited reads in SSWD-affected tissues may also relate to the proportion of contaminating co-extracted host nucleic acids in SSWD-affected and grossly normal specimens (i.e., viral sequence space vs. host and other contaminant sequence space). Total extracted DNA quantities decrease in affected specimens [20]. Viral nucleic acids, which may be more protected from enzymatic decay within capsids than host RNAs, may become more pronounced when host tissues degrade. Hence, viral nucleic acids may recruit more regularly to viral genome fragments solely because they comprise a greater proportion of virome sequence space.

## 4. Conclusions

Our results illustrate that both grossly normal and SSWD-affected asteroids are associated with RNA viruses which are similar to those recovered in metagenomic and metatranscriptomic surveys of marine invertebrates performed elsewhere. SSWD in *Pisaster ochraceus* is not associated with any specific viral genotype detected in this survey. Rather, wasting is associated with an increased richness of viral genome fragments recruiting reads in affected tissues, which may be due to factors influencing their replication or due to the balance between host and viral RNA in tissues. This work provides additional evidence that densoviruses, and particularly the Asteroid ambidensovirus 1 is not consistently associated with sea star wasting, and emphasizes the lack of association more generally between viruses and SSWD. Our work raises interesting questions about the influence of environmental factors in influencing the replication and perhaps pathology of viruses in marine diseases.

## Figures and Tables

**Table 1:** Viral metagenomics library characteristics for *Pisaster ochraceus* wasting temporal assay.

Library Name	Specimen Name	Date	Tissue Type	Library Size (Reads)	Reads Matching Viruses	BioSample Accession
SC1	SSWD-affected 1	22 July 2018	Control	3,658,045	554,809	SRR11931187
SC2	SSWD-affected 1	26 July 2018	Scar	4,099,676	582,091	SRR11931186
SC3	SSWD-affected 1	26 July 2018	Control	3,592,838	597,395	SRR11931179
SC4	SSWD-affected 1	26 July 2018	Lesion	3,255,385	169,582	SRR11931178
SC5	SSWD-affected 1	27 July 2018	Lesion	3,443,200	871,889	SRR11931177
SC6	SSWD-affected 2	22 July 2018	Control	4,948,712	1,007,543	SRR11931176
SC7	SSWD-affected 2	4 August 2018	Control	2,703,665	624,989	SRR11931175
SC8	SSWD-affected 2	4 August 2018	Scar	2,797,069	507,994	SRR11931174
SC9	SSWD-affected 2	4 August 20188	Lesion	3,678,615	1,306,746	SRR11931173
SC10	SSWD-affected 2	6 August 2018	Lesion	3,259,328	924,368	SRR11931172
SC11	Grossly Normal 1	22 July 2018	Control	2,954,408	2349	SRR11931185
SC12	Grossly Normal 1	9 August 2018	Control	1,919,780	2694	SRR11931184
SC13	Grossly Normal 1	9 August 2018	Scar	1,939,732	3134	SRR11931183
SC14	Grossly Normal 2	22 July 2018	Control	3,273,980	3851	SRR11931182
SC15	Grossly Normal 2	9 August 2018	Control	1,595,206	1112	SRR11931181
SC16	Grossly Normal 2	9 August 2018	Scar	1,196,532	882	SRR11931180

Control = grossly normal tissue; Scar = artificial scar margin; Lesion = wasting lesion margin.

**Table 2:** Fisher’s exact test results for viral contigs comparing condition (SSWD-affected vs. grossly normal), sampling time (initial, time of lesion formation, time of death or experiment termination), and sample tissue type (control, artificial scar, wasting lesion). Only contigs returning any significant (*p* < 0.05) result are reported.

Contig #	*p*-Value		
	Condition	Time	Sample Type
Contig 2401	ns	0.023	Ns
Contig 12092	0.001	ns	Ns
Contig 12093	0.003	0.022	Ns

## Supplementary Materials

The following are available online at https://www.mdpi.com/1999-4915/12/11/1332/s1, Figure S1: Sampling schema for observation of viromes in control, artificial scar, and wasting lesion margins, Figure S2: Coverage plots for all viral contigs retrieved in this survey, Figure S3: Phylogenetic reconstruction of *Picornavirales*-like genome fragments by Maximum Likelihood, Figure S4: Nodavirus-like genome fragment contig map, Figure S5: Phylogenetic reconstruction of *Nodamuravirales*-like genome fragments by Maximum Likelihood, Figure S6: Phylogenetic reconstruction of *Piccovirales*-like genome fragments by Maximum Likelihood.

## References

[1] Mead A.D. (1898). Twenty-Eighth Annual Report of the Commissioners of Inland Fisheries, Made to the General Assembly at Its January Session, 1898.

[2] Hewson I., Button J.B., Gudenkauf B.M., Miner B., Newton A.L., Gaydos J.K., Wynne J., Groves C.J., Hendler G., Murray M. (2014). Densovirus associated with sea-star wasting disease and mass mortality. Proc. Natl. Acad. Sci. USA.

[3] Hewson I., Sullivan B., Jackson E.W., Xu Q., Long H., Lin C., Quijano Cardé E.M., Seymour J., Siboni N., Jones M.R.L. (2019). Perspective: Something old, something new? Review of wasting and other mortality in Asteroidea (Echinodermata). Front. Mar. Sci..

[4] Bucci C., Francoeur M., McGreal J., Smolowitz R., Zazueta-Novoa V., Wessel G.M., Gomez-Chiarri M. (2017). Sea star wasting disease in *Asterias forbesi* along the Atlantic coast of North America. PLoS ONE.

[5] Hewson I., Bistolas K.S.I., Quijano Carde E.M., Button J.B., Foster P.J., Flanzenbaum J.M., Kocian J., Lewis C.K. (2018). Investigating the complex association between viral ecology, environment and Northeast Pacific sea star wasting. Front. Mar. Sci..

[6] Jackson E.W., Wilhelm R.C., Johnson M.R., Lutz H.L., Danforth I., Gaydos J.K., Hart M.W., Hewson I. (2020). Diversity of sea star-associated densoviruses and transcribed endogenized viral elements of densovirus origin. J. Virol..

[7] Jackson E.W., Pepe-Ranney C., Johnson M.R., Distel D.L., Hewson I. (2020). A highly prevalent and pervasive densovirus discovered among sea stars from the North American Atlantic coast. Appl. Environ. Microbiol..

[8] Aquino C.A., Besemer R.M., DeRito C.M., Kocian J., Porter I.R., Raimondi P.T., Rede J.E., Schiebelhut L.M., Sparks J.P., Wares J.P. (2020). Evidence that non-pathogenic microorganisms drive sea star wasting disease through boundary layer oxygen diffusion limitation. bioRxiv.

[9] Aalto E.A., Lafferty K.D., Sokolow S.H., Grewelle R.E., Ben-Horin T., Boch C.A., Raimondi P.T., Bograd S.J., Hazen E.L., Jacox M.G. (2020). Models with environmental drivers offer a plausible mechanism for the rapid spread of infectious disease outbreaks in marine organisms. Sci. Rep..

[10] Eisenlord M.E., Groner M.L., Yoshioka R.M., Elliott J., Maynard J., Fradkin S., Turner M., Pyne K., Rivlin N., van Hooidonk R. (2016). Ochre star mortality during the 2014 wasting disease epizootic: Role of population size structure and temperature. Phil. Trans. R. Soc. B.

[11] Menge B.A., Cerny-Chipman E.B., Johnson A., Sullivan J., Gravem S., Chan F. (2016). Sea star wasting disease in the keystone predator *Pisaster ochraceus* in Oregon: Insights into differential population impacts, recovery, predation rate, and temperature effects from long-term research. PLoS ONE.

[12] Lloyd M.M., Pespeni M.H. (2018). Microbiome shifts with onset and progression of Sea Star Wasting Disease revealed through time course sampling. Sci. Rep..

[13] Newton A.L., Smolowitz R., Terio K.A., McAloose D., Leger J.S. (2018). Chapter 41—Invertebrates. Pathology of Wildlife and Zoo Animals.

[14] Nuñez-Pons L., Work T.M., Angulo-Preckler C., Moles J., Avila C. (2018). Exploring the pathology of an epidermal disease affecting a circum-Antarctic sea star. Sci. Rep..

[15] Thurber R.V., Haynes M., Breitbart M., Wegley L., Rohwer F. (2009). Laboratory procedures to generate viral metagenomes. Nat. Protoc..

[16] Ng F.F.T., Wheeler E., Greig D., Waltzek T.B., Gulland F., Breitbart M. (2011). Metagenomic identification of a novel anellovirus in Pacific harbor seal (*Phoca vitulina richardsii*) lung samples and its detection in samples from multiple years. J. Gen. Virol..

[17] Hewson I., Johnson M.R., Tibbetts I.R. (2020). An unconventional flavivirus and other RNA viruses in the sea cucumber (Holothuroidea; Echinodermata) virome. Viruses.

[18] Altschul S.F., Gish W., Miller W., Myers E.W., Lipman D.J. (1990). Basic local alignment search tool. J. Mol. Biol..

[19] Altschul S.F., Madden T.L., Schaffer A.A., Zhang J., Zhang Z., Miller W., Lipman D.J. (1997). Gapped BLAST and PSI-BLAST: A new generation of protein database search programs. Nucleic Acids Res..

[20] Hewson I. (2019). Technical pitfalls that bias comparative microbial community analyses of aquatic disease. Dis. Aquat. Org..

[21] Kumar G., Li M., Knyaz C., Tamura K. (2018). MEGAX: Molecular evolutionary genetics analysis across computing platforms. Molec. Biol. Evol..

[22] Shi M., Lin X.-D., Tian J.-H., Chen L.-J., Chen X., Li C.-X., Qin X.-C., Li J., Cao J.-P., Eden J.-S. (2016). Redefining the invertebrate RNA virosphere. Nature.

[23] Shi M., Lin X.-D., Vasilakis N., Tian J.-H., Li C.-X., Chen L.-J., Eastwood G., Diao X.-N., Chen M.-H., Chen X. (2016). Divergent viruses discovered in arthropods and vertebrates revise the evolutionary history of the *Flaviviridae* and related viruses. J. Virol..

[24] Wille M., Eden J.-S., Shi M., Klaassen M., Hurt A.C., Holmes E.C. (2018). Virus–virus interactions and host ecology are associated with RNA virome structure in wild birds. Mol. Ecol..

[25] Debat H.J. (2017). An RNA virome associated to the golden orb-weaver spider *Nephila clavipes*. Front. Microbiol..

[26] Shi M., White V.L., Schlub T., Eden J.-S., Hoffmann A.A., Holmes E.C. (2018). No detectable effect of *Wolbachia* on the prevalence and abundance of the RNA virome of *Drosophila melanogaster*. Proc. R. Soc. B.

[27] Niu J., Li X.-L., Wu Y.-L., Sun Q.-Z., Zhang W., Cao M., Wang J.-J. (2020). RNA virome screening in diverse but ecologically related citrus pests reveals potential virus-host interactions. J. Invertebr. Pathol..

[28] Ott Rutar S., Kordis D. (2020). Analysis of the RNA virome of basal hexapods. PeerJ.

[29] Wille M., Harvey E., Shi M., Gonzalez-Acuña D., Holmes E.C., Hurt A.C. (2020). Sustained RNA virome diversity in Antarctic penguins and their ticks. ISME J..

[30] Pettersson J.H.O., Shi M., Eden J.-S., Holmes E.C., Hesson J.C. (2019). Meta-Transcriptomic comparison of the RNA viromes of the mosquito vectors *Culex pipiens* and *Culex torrentium* in northern Europe. Viruses.

[31] Mahar J.E., Shi M., Hall R.N., Strive T., Holmes E.C. (2020). Comparative analysis of RNA virome composition in rabbits and associated ectoparasites. J. Virol..

[32] Öhlund P., Hayer J., Lundén H., Hesson J.C., Blomström A.-L. (2019). Viromics reveal a number of novel RNA viruses in swedish mosquitoes. Viruses.

[33] Bennett A.J., Bushmaker T., Cameron K., Ondzie A., Niama F.R., Parra H.-J., Mombouli J.-V., Olson S.H., Munster V.J., Goldberg T.L. (2019). Diverse RNA viruses of arthropod origin in the blood of fruit bats suggest a link between bat and arthropod viromes. Virology.

[34] Hewson I., Bistolas K.S.I., Button J.B., Jackson E.W. (2018). Occurrence and seasonal dynamics of RNA viral genotypes in three contrasting temperate lakes. PLoS ONE.

[35] Miranda J.A., Culley A.I., Schvarcz C.R., Steward G.F. (2016). RNA viruses as major contributors to Antarctic virioplankton. Environ. Microbiol..

[36] Lang A.S., Rise M.L., Culley A.I., Steward G.F. (2009). RNA viruses in the sea. FEMS Microbiol. Rev..

[37] Culley A.I., Lang A.S., Suttle C.A. (2007). The complete genomes of three viruses assembled from shotgun libraries of marine RNA virus communities. Virol. J..

[38] Culley A.I., Steward G.F. (2007). New genera of RNA viruses in subtropical seawater, inferred from polymerase gene sequences. Appl. Environ. Microbiol..

[39] Culley A.I., Lang A.S., Suttle C.A. (2003). High diversity of unknown picorna-like viruses in the sea. Nature.

[40] Culley A.I., Lang A.S., Suttle C.A. (2006). Metagenomic analysis of coastal RNA virus communities. Science.

[41] Rosani U., Gerdol M. (2017). A bioinformatics approach reveals seven nearly-complete RNA-virus genomes in bivalve RNA-seq data. Virus Res..

[42] Moniruzzaman M., Wurch L.L., Alexander H., Dyhrman S.T., Gobler C.J., Wilhelm S.W. (2016). Virus-host infection dynamics of marine single-celled eukaryotes resolved from metatranscriptomics. bioRxiv.

[43] Sakuna K., Elliman J., Owens L. (2017). Discovery of a novel *Picornavirales*, *Chequa iflavirus*, from stressed redclaw crayfish (*Cherax quadricarinatus*) from farms in northern Queensland, Australia. Virus Res..

[44] Lachnit T., Thomas T., Steinberg P. (2016). Expanding our understanding of the seaweed holobiont: RNA viruses of the red alga *Delisea pulchra*. Front. Microbiol..

[45] Tai V., Lawrence J.E., Lang A.S., Chan A.M., Culley A.I., Suttle C.A. (2003). Characterization of HaRNAV, a single-stranded RNA virus causing lysis of *Heterosigma akashiwo* (*Raphidophyceae*). J. Phycol..

[46] Toyoda K., Kimura K., Osada K., Williams D.M., Adachi T., Yamada K., Tomaru Y. (2019). Novel marine diatom ssRNA virus NitRevRNAV infecting *Nitzschia reversa*. Plant Ecol. Evol..

[47] Bonami J.R., Hasson K.W., Mari J., Poulos B.T., Lightner D.V. (1997). Taura syndrome of marine penaeid shrimp: Characterization of the viral agent. J. Gen. Virol..

[48] Munday B.L., Kwang J., Moody N. (2002). Betanodavirus infections of teleost fish: A review. J. Fish Dis..

[49] Yong C.Y., Yeap S.K., Omar A.R., Tan W.S. (2017). Advances in the study of nodavirus. PeerJ.

[50] Jean-Michel A., François H., Donald V.L., Rita M.R., Jocelyne M., Jean-Robert B. (1999). A viral disease associated with mortalities in hatchery-reared postlarvae of the giant freshwater prawn *Macrobrachium rosenbergii*. Dis. Aquat. Org..

[51] Gomez D.K., Baeck G.W., Kim J.H., Choresca C.H., Park S.C. (2008). Molecular detection of betanodaviruses from apparently healthy wild marine invertebrates. J. Invertebr. Pathol..

[52] Gomez D.K., Lim D.J., Baeck G.W., Youn H.J., Shin N.S., Youn H.Y., Hwang C.Y., Park J.H., Park S.C. (2006). Detection of betanodaviruses in apparently healthy aquarium fishes and invertebrates. J. Vet. Sci..

[53] Ng T.F.F., Alavandi S., Varsani A., Burghart S., Breitbart M. (2013). Metagenomic identification of a nodavirus and a circular ssDNA virus in semi-purified viral nucleic acids from the hepatopancreas of healthy *Farfantepenaeus duorarum* shrimp. Dis. Aquat. Org..

[54] Gomez D.K., Baeck G.W., Kim J.H., Choresca C.H., Park S.C. (2008). Genetic analysis of betanodaviruses in subclinically infected aquarium fish and invertebrates. Curr. Microbiol..

[55] Bergoin M., Tijssen P. (2000). Molecular biology of *Densovirinae*. Contrib. Microbiol..

[56] Tijssen P., Bergoin M. (1995). Densonucleosis viruses constitute an increasingly diversified subfamily among the parvoviruses. Semin. Virol..

[57] Kang Y.J., Huang W., Zhao A.L., Lai D.D., Shao L., Shen Y.Q., Deng X., Delwart E., Zhang W. (2017). Densoviruses in oyster *Crassostrea ariakensis*. Arch. Virol..

[58] Richard J.C., Leis E., Dunn C.D., Agbalog R., Waller D., Knowles S., Putnam J., Goldberg T.L. (2020). Mass mortality in freshwater mussels (*Actinonaias pectorosa*) in the Clinch River, USA, linked to a novel densovirus. Sci. Rep..

[59] Gudenkauf B.M., Hewson I. (2016). Comparative metagenomics of viral assemblages inhabiting four phyla of marine invertebrates. Front. Mar. Sci..

[60] Bochow S., Condon K., Elliman J., Owens L. (2015). First complete genome of an Ambidensovirus; *Cherax quadricarinatus* densovirus, from freshwater crayfish *Cherax quadricarinatus*. Mar. Genom..

[61] Fargette D., Pinel A., Abubakar Z., Traoré O., Brugidou C., Fatogoma S., Hébrard E., Choisy M., Séré Y., Fauquet C. (2004). Inferring the evolutionary history of Rice Yellow Mottle Virus from genomic, phylogenetic, and phylogeographic studies. J. Virol..

[62] Tamm T., Truve E. (2000). Sobemoviruses. J. Virol..

[63] Laffy P.W., Botté E.S., Wood-Charlson E.M., Weynberg K.D., Rattei T., Webster N.S. (2019). Thermal stress modifies the marine sponge virome. Environ. Microbiol. Rep..

[64] Grasis J.A., Lachnit T., Anton-Erxleben F., Lim Y.W., Schmieder R., Fraune S., Franzenburg S., Insua S., Machado G., Haynes M. (2014). Species-specific viromes in the ancestral holobiont *Hydra*. PLoS ONE.

[65] Thurber R.L.V., Barott K.L., Hall D., Liu H., Rodriguez-Mueller B., Desnues C., Edwards R.A., Haynes M., Angly F.E., Wegley L. (2008). Metagenomic analysis indicates that stressors induce production of herpes-like viruses in the coral *Porites compressa*. Proc. Natl. Acad. Sci. USA.

[66] Frakolaki E., Kaimou P., Moraiti M., Kalliampakou K.I., Karampetsou K., Dotsika E., Liakos P., Vassilacopoulou D., Mavromara P., Bartenschlager R. (2018). The role of tissue oxygen tension in Dengue virus replication. Cells.

[67] Moon E.J., Jeong C.H., Jeong J.W., Kim K.R., Yu D.Y., Murakami S., Kim C.W., Kim K.W. (2004). Hepatitis B virus X protein induces angiogenesis by stabilizing hypoxia-inducible factor-1alpha. FASEB J..

[68] Wakisaka N., Kondo S., Yoshizaki T., Murono S., Furukawa M., Pagano J.S. (2004). Epstein-Barr virus latent membrane protein 1 induces synthesis of hypoxia-inducible factor 1α. Mol. Cell. Biol..

[69] Ruan H., Su H., Hu L., Lamborn K.R., Kan Y.W., Deen D.F. (2001). A hypoxia-regulated adeno-associated virus vector for cancer-specific gene therapy. Neoplasia.

[70] Aghi M.K., Liu T.-C., Rabkin S., Martuza R.L. (2009). Hypoxia enhances the replication of oncolytic Herpes Simplex virus. Mol. Ther..

[71] Jiang J.-H., Wang N., Li A., Liao W.-T., Pan Z.-G., Mai S.-J., Li D.-J., Zeng M.-S., Wen J.-M., Zeng Y.-X. (2006). Hypoxia can contribute to the induction of the Epstein-Barr virus (EBV) lytic cycle. J. Clin. Virol..

[72] Gan E.S., Cheong W.F., Chan K.R., Ong E.Z., Chai X., Tan H.C., Ghosh S., Wenk M.R., Ooi E.E. (2017). Hypoxia enhances antibody-dependent dengue virus infection. EMBO J..

