# Supplementary material for: Virome Variation during Sea Star Wasting Disease Progression in Pisaster ochraceus (Asteroidea, Echinodermata)
Source: Viruses. 2020 Nov 20;12(11):1332. doi: 10.3390/v12111332 (PMC7699681; doi:10.3390/v12111332)
Supplement: Supplementary file 1 [file viruses-12-01332-s001.zip › Hewson_FigS1_FINAL.pdf]

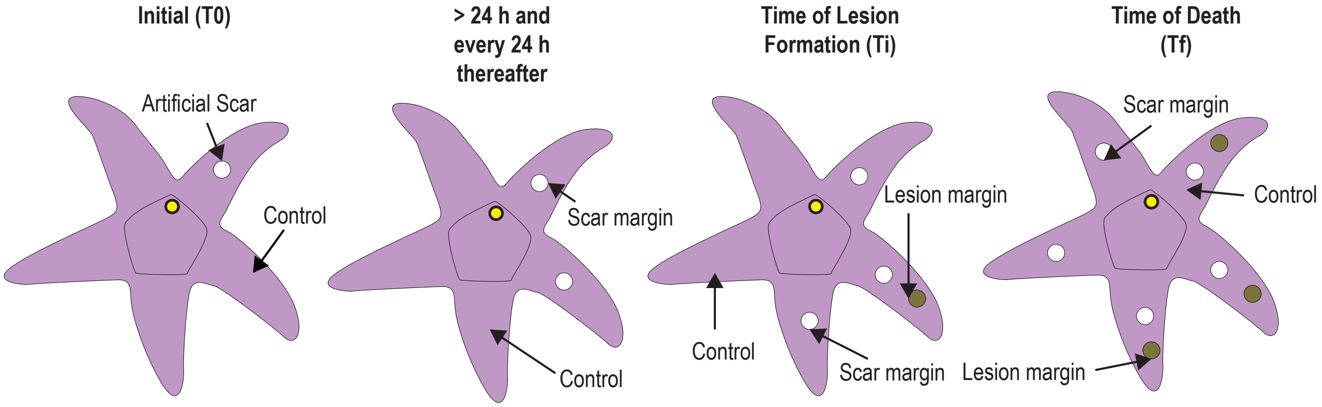

Figure S1: After 48 h acclimation, a small scar (~ 5 mm long) was made on a single ray of each specimen using a sterile 4 mm biopsy punch. Individual asteroids were monitored daily for the presence of lesions (which were defined as non-focal loss of epidermal tissues revealing the underlying body wall; see Fig. 1). After 72 h, artificial scar margin tissues (~ 3mm x 2mm) were collected using sterile 4mm biopsy punches. New artificial scars were made on adjacent rays each day, and sampled after each 24 h period. When wasting lesions were observed (the first wasting lesion was observed at 96 h), their margins were sampled following the same approach, and grossly normal tissues well away (> 1cm) from the lesion collected at the same time for comparison.
