# Supplementary material for: Virome Variation during Sea Star Wasting Disease Progression in Pisaster ochraceus (Asteroidea, Echinodermata)
Source: Viruses. 2020 Nov 20;12(11):1332. doi: 10.3390/v12111332 (PMC7699681; doi:10.3390/v12111332)
Supplement: Supplementary file 1 [file viruses-12-01332-s001.zip › Hewson_FigS2_FINAL.pdf]

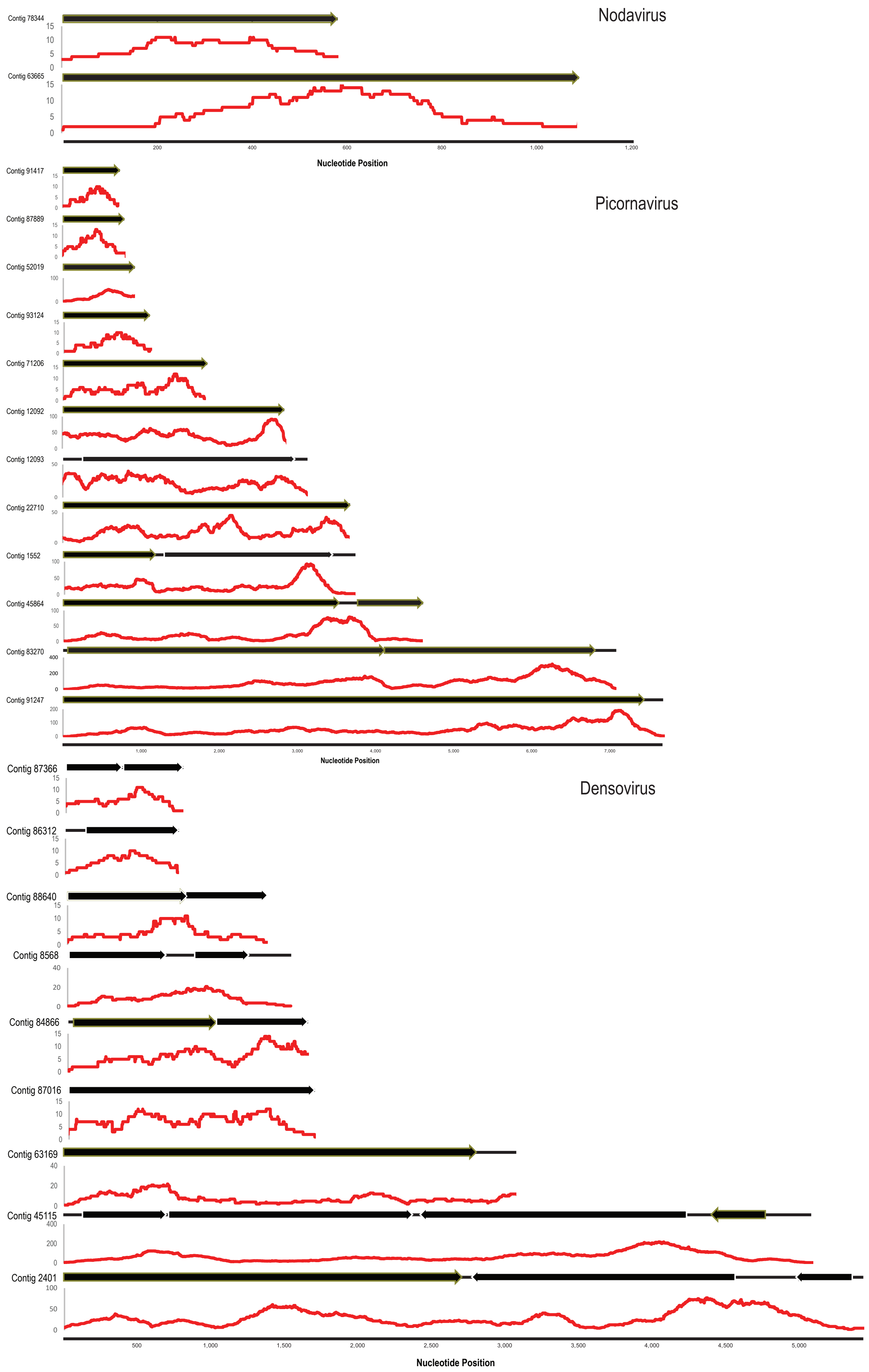

Figure S2: Coverage plots for each contig retrieved in this survey. Coverage was assessed by read mapping in the CLC Genomics Workbench 4.0 using a 0.2 read length cutoff and 0.95 similarity. The total contig length is indicated by the solid black line running through arrows (ORFs). Note difference in scale between viral orders.
