# Supplementary material for: Virome Variation during Sea Star Wasting Disease Progression in Pisaster ochraceus (Asteroidea, Echinodermata)
Source: Viruses. 2020 Nov 20;12(11):1332. doi: 10.3390/v12111332 (PMC7699681; doi:10.3390/v12111332)
Supplement: Supplementary file 1 [file viruses-12-01332-s001.zip › Hewson_FigS3_FINAL.pdf]

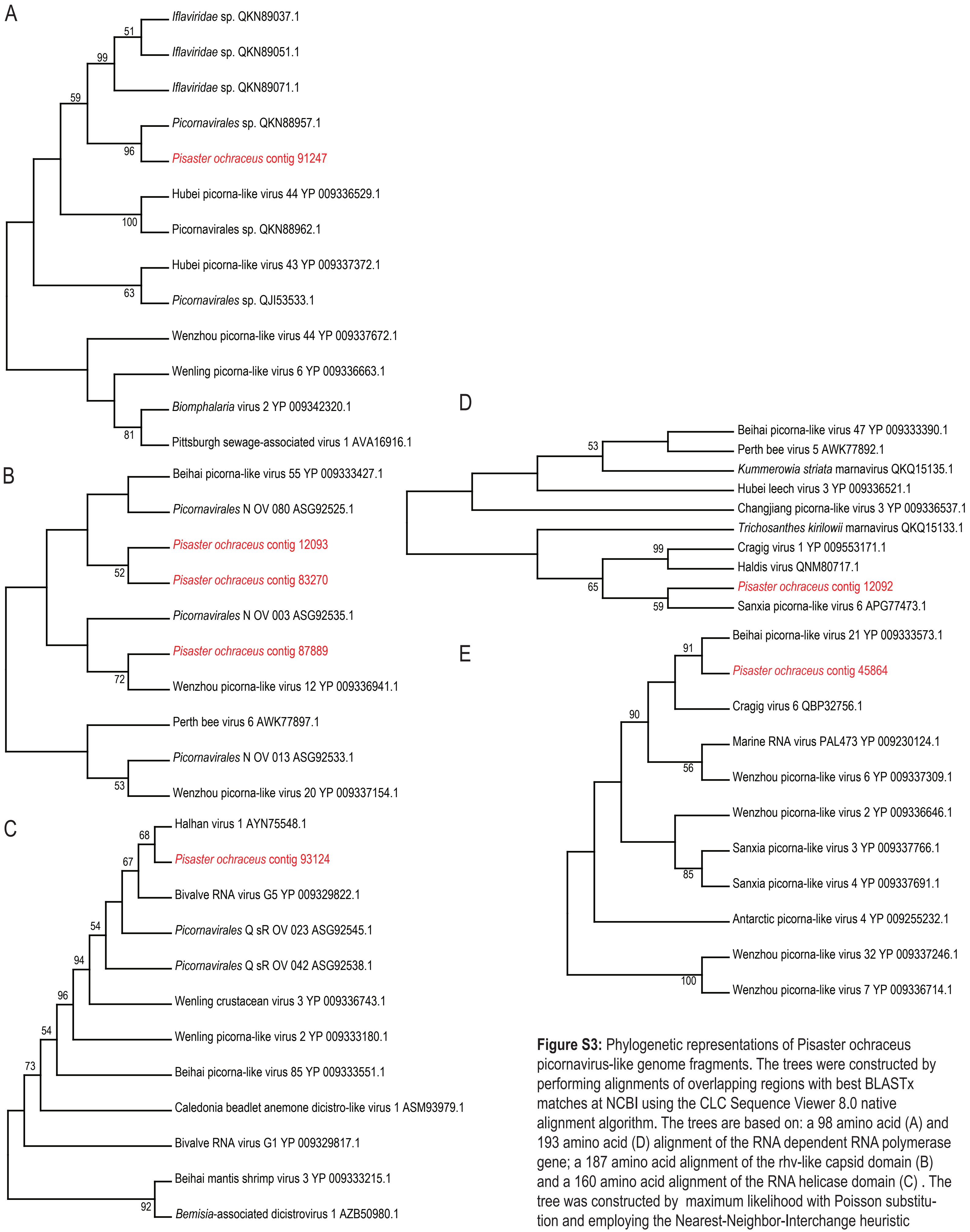

Figure S3: Phylogenetic representations of Pisaster ochraceus picornavirus-like genome fragments. The trees were constructed by performing alignments of overlapping regions with best BLASTx matches at NCBI using the CLC Sequence Viewer 8.0 native alignment algorithm. The trees are based on: a 98 amino acid (A) and 193 amino acid (D) alignment of the RNA dependent RNA polymerase gene; a 187 amino acid alignment of the rhv-like capsid domain (B) and a 160 amino acid alignment of the RNA helicase domain (C) . The tree was constructed by maximum likelihood with Poisson substitution and employing the Nearest-Neighbor-Interchange heuristic method in MEGA 6.0.
