# Supplementary material for: Virome Variation during Sea Star Wasting Disease Progression in Pisaster ochraceus (Asteroidea, Echinodermata)
Source: Viruses. 2020 Nov 20;12(11):1332. doi: 10.3390/v12111332 (PMC7699681; doi:10.3390/v12111332)
Supplement: Supplementary file 1 [file viruses-12-01332-s001.zip › Hewson_FigS4_FINAL.pdf]

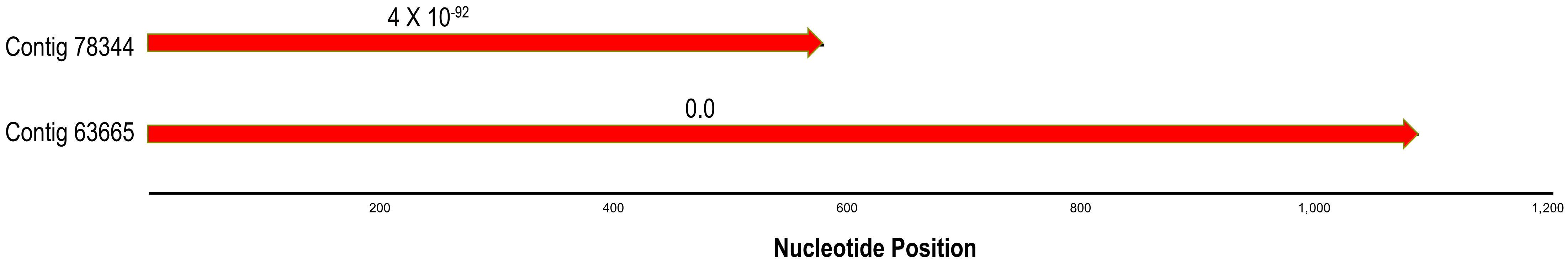

Figure S4: Maps of contiguous sequences matching Nodamuravirales recovered in this survey of Pisaster ochraceus. Contigs were annotated based on BLASTx (e-value < 1 x 10-20) against the non-redundant database at NCBI. All matches were to the RNA1 genome segment of nodaviruses. Numbers above the ORFs indicate the e-value of BLAST results. The total contig lengths are indicated by solid lines running through and between ORFs.
