# Supplementary material for: Virome Variation during Sea Star Wasting Disease Progression in Pisaster ochraceus (Asteroidea, Echinodermata)
Source: Viruses. 2020 Nov 20;12(11):1332. doi: 10.3390/v12111332 (PMC7699681; doi:10.3390/v12111332)
Supplement: Supplementary file 1 [file viruses-12-01332-s001.zip › Hewson_FigS5_FINAL.pdf]

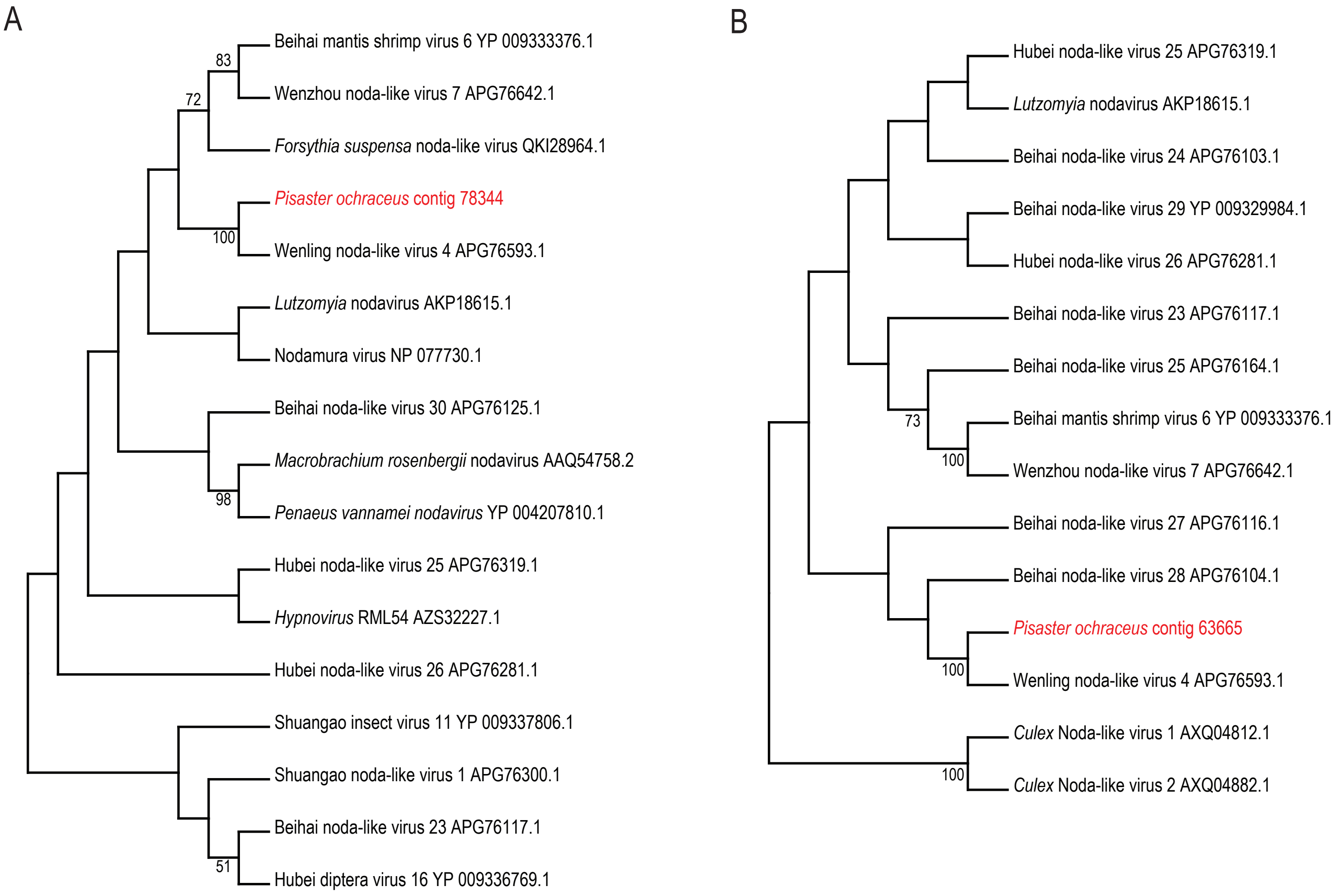

Figure S5: Phylogenetic representations of Pisaster ochraceus nodavirus-like genome fragments. The trees were constructed by performing alignments of overlapping regions with best BLASTx matches at NCBI using the CLC Sequence Viewer 8.0 native alignment algorithm. The trees were constructed based on a 101 amino acid (A) and 559 amino acid (B) alignments of the RNA dependent RNA polymerase gene of the nodavirus RNA1 genome fragment including best matches at NCBI. The tree was constructed by maximum likelihood with Poisson substitution and employing the Nearest-Neighbor-Interchange heuristic method in MEGA 6.0.
