# Supplementary material for: Virome Variation during Sea Star Wasting Disease Progression in Pisaster ochraceus (Asteroidea, Echinodermata)
Source: Viruses. 2020 Nov 20;12(11):1332. doi: 10.3390/v12111332 (PMC7699681; doi:10.3390/v12111332)
Supplement: Supplementary file 1 [file viruses-12-01332-s001.zip › Hewson_FigS6_FINAL.pdf]

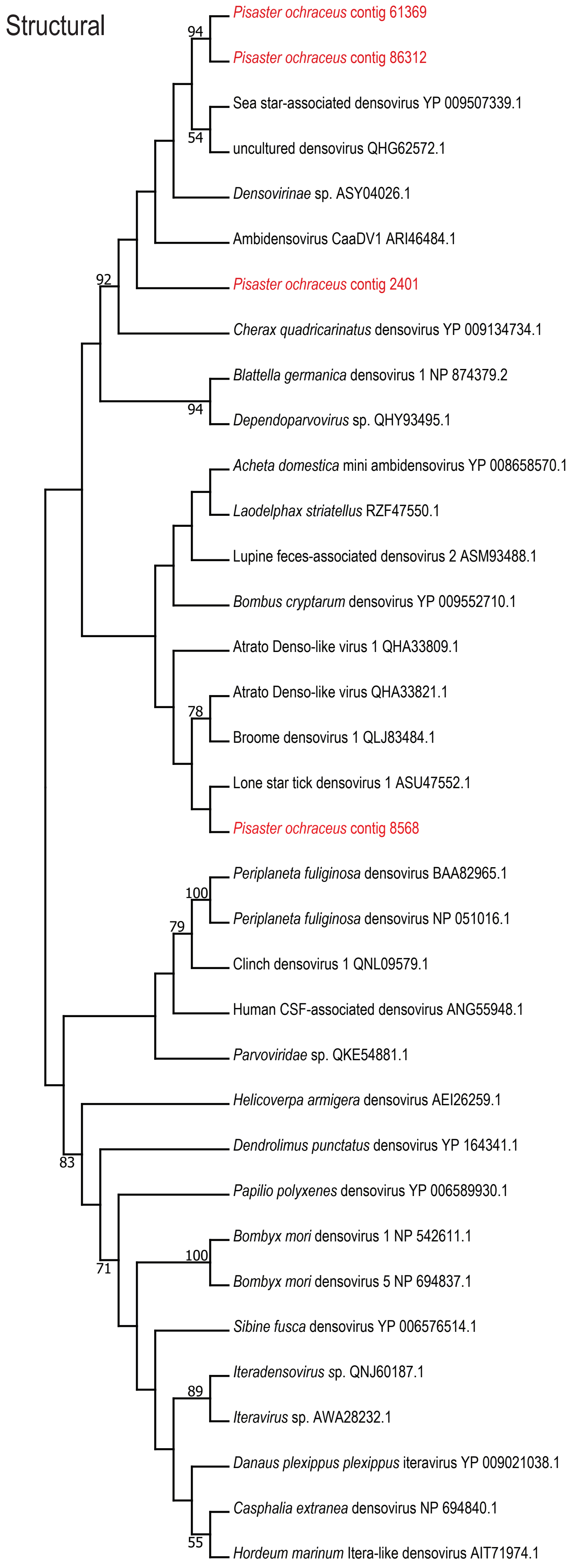

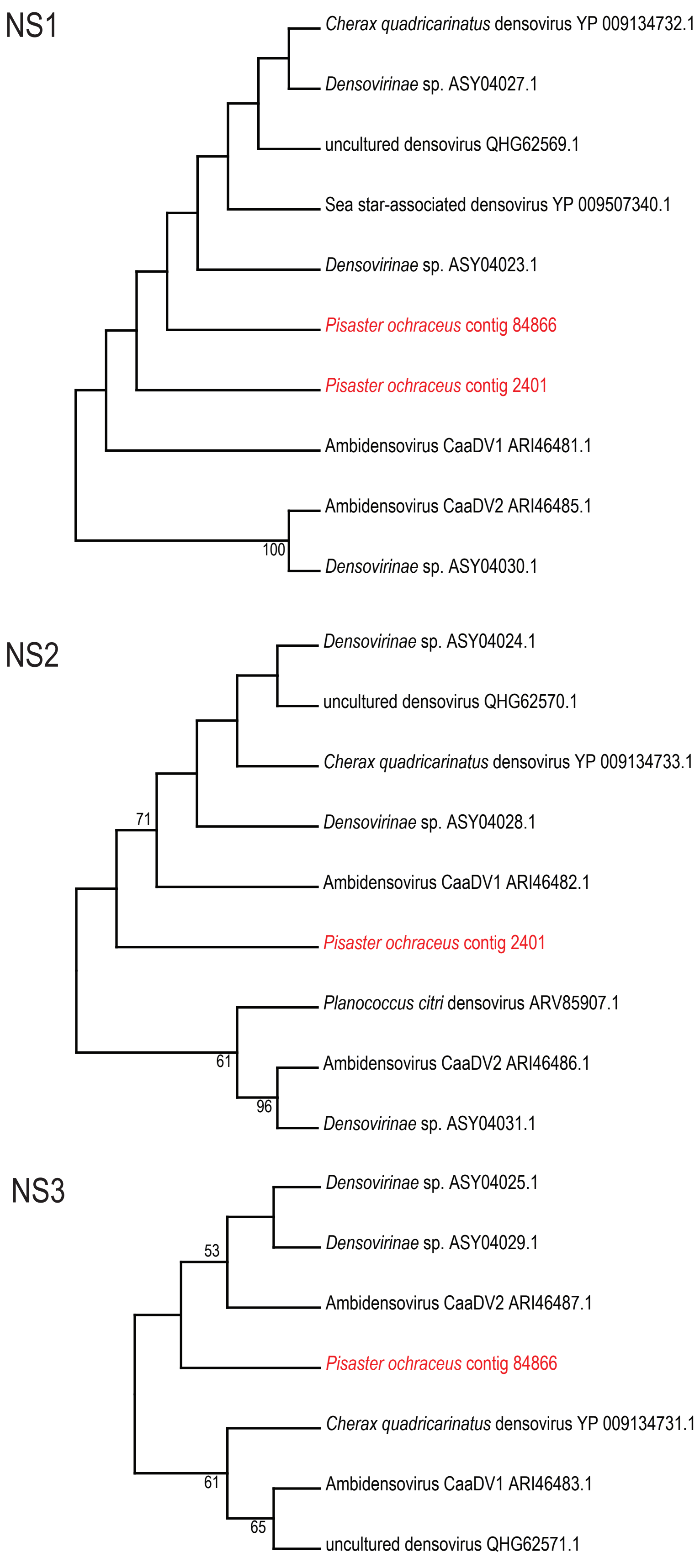

Figure S6: Phylogenetic representations of Pisaster ochraceus densovirus-like genome fragments. The trees were constructed based on: a 103 amino acid alignment of the structural (coat protein) gene; a 83 amino acid of the non-structural 1 (NS1) gene; a 112 amino acid of the NS2 gene; and a 111 amino acid of the NS3 gene. Phylogenetic representations include best matches by BLASTx against the non-redundant database at NCBI. The tree was constructed by maximum likelihood with Poisson substitution and employing the Nearest-Neighbor-Interchange heuristic method in MEGA 6.0.
